# A simulation study on enhancing sterilization efficiency in medical plastics through gamma radiation optimization

**DOI:** 10.1038/s41598-023-47771-9

**Published:** 2023-11-20

**Authors:** Xin Yuan, Fang Liu, Hongchi Zhou, Bin Liu, Guanda Li, Peiguang Yan, Guoping Li, Xiaoru Luo, Xuefeng Lyu, Jinxing Cheng, Fenglei Niu

**Affiliations:** 1 Beijing Key Laboratory of Passive Safety Technology for Nuclear Energy, School of Nuclear Science and Engineering, North China Electric Power University, Beijing, 102206 China; 2 Shenzhen Key Laboratory of Laser Engineering, College of Physics and Optoelectronic Engineering, Shenzhen University, Shenzhen, 518060 China; 3 The Institute of NBC Defense, Chinese PLA Army, Beijing, 102205 China; 4 Institute of Nuclear and New Energy Technology, Tsinghua University, Beijing, 100084 China

**Keywords:** Biophysics, Environmental sciences, Environmental social sciences, Nuclear organization, Nuclear transport, Fungi, Policy and public health in microbiology, Virology

## Abstract

Gamma radiation is progressively emerging as an effective method to enhance the sterilization efficiency of medical plastics including Polyvinyl chloride (PVC). The parameters of the radiation facility will affect the efficiency of radiation sterilization. To investigate these effects, we simulate the gamma radiation sterilization performance of PVC material sample using Monte Carlo Method. The simulation results indicated that compared with the sterilization time of 20–90 min from high-temperature steam sterilization of medical waste, by optimizing the parameters of the model radiation facility, the radiation sterilization time can be reduced to 6.61 min. The optimized model facility parameters are as follows: the gamma photon energy is 1.25 MeV, the model space is 300 × 300 × 300 cm^3^, the reflective layer material is concrete and its thickness is 8 cm, the PVC sample layer area is 100 × 100 cm^2^, the distance between the radiation source and the PVC sample layer is 150 cm, the energy deposition in the bottom layer of the PVC sample layer is 1.31315 × 10^–6^ MeV/g. This study offers a potentially feasible way for PVC sterilization, while also providing a crucial reference for the further promotion and application of radiation sterilization technology.

## Introduction

According to reports from the Environmental Protection Agency (EPA), only 7% of plastic waste generated annually is recycled, approximately 8% is incinerated, and the remainder is landfilled^1^. The widespread demand for medical plastic products coupled with the issue of low recycling rates have led to significant environmental pollution^1^. To solve this problem, the sterilization efficiency of medical plastic products needs to be further improved. PVC, as a primary material in medical plastic products, is extensively used in the field of medical devices^2–4^. However, a substantial amount of used medical PVC products are either incinerated or disposed of in sanitary landfills, exacerbating environmental pollution^5^. Especially during the spread of an epidemic, there is a significant surge in demand for PVC products^6,7^, resulting in a corresponding rapid increase in the quality of medical waste. Reports indicate that on February 24, 2020, Wuhan City generated 200 tons of clinical waste^5^. The King Abdullah University Hospital in Jordan found that coronavirus patients generate 14.16 kg of medical waste per day^8^. Failure to effectively manage such medical waste could pose serious environmental hazards. Confronted with the stark problem of rapidly increasing medical waste and an acute shortage of medical plastic products, the sterilization and recycling of medical plastic products still face formidable challenges^9,10^.

Currently, traditional sterilization methods such as chemical disinfection, high-temperature steam sterilization, and electromagnetic wave sterilization method have limitations in the field of sterilization^11–13^. Chemical disinfection methods result in a significant accumulation of chemical residues, while high-temperature steam sterilization may lead to high energy consumption, long sterilization times, the generation of toxic gases, and material deformation issues. Electromagnetic wave sterilization incurs high operational costs and leads to the production of toxic gases^13^. Furthermore, incineration and sanitary landfilling struggle to effectively address the treatment of medical plastic products^14^. Incineration not only pollutes the environment but also releases toxic halogenated compounds during thermal degradation, causing harm to the lungs and hearts of humans^15^. Due to the stable molecular structure of PVC products, they are resistant to corrosion or decomposition during natural degradation processes^5^. Sanitary landfilling consumes extensive land resources. Furthermore, prolonged natural degradation can also produce toxic gases, endangering plants, animals, aquatic organisms, and humans^14^.

To respond these challenges, the World Health Organization (WHO) has issued a call to explore a new environmentally friendly and efficient sterilization technology^16^. The United States Food and Drug Administration (FDA) has proposed the use of gamma radiation for sterilizing disposable medical devices, initiating a pilot program for radiation sterilization^17^. Furthermore, they have updated the Recognized Consensus Standards database to endorse the application of radiation sterilization technology in medical devices^18^.

Radiation sterilization technology, as an advanced, no harmful gas generation, and efficient sterilization method, is gaining increasing popularity among the public. This technology utilizes electron beams, neutron beams, or gamma rays to comprehensively eliminate bacteria, viruses, and other microorganisms on the surface and interior of medical plastic products, ensuring the safety of recycling. Compared to traditional methods, radiation sterilization technology is pollution-free, rapid, efficient, and leaves no residue^19^. It provides a novel solution for the sterilization and recycling of medical plastic products. Currently, there have been relevant studies on the radiation sterilization of medical devices. For example, Josef Mittendorfer used 10 MeV electron beam radiation for sterilizing medical devices^20^, as well as the gamma rays and electron beam radiation sterilization of plastic packaging products^21^. Previous research has indicated that the tensile yield strength of PVC material changes less under low doses of gamma radiation^22^.

This study aims to investigate the impact of irradiation facility parameters on the efficiency of irradiation sterilization of PVC materials. By delving into the principles and characteristics of radiation and analyzing the influence factors, optimal gamma radiation facility parameters are identified. Subsequently, simulation calculations are conducted to determine the photon energy deposition in the PVC sample layer. Based on the radiation sterilization dosage, the radiation sterilization time can be calculated. This study offers a potentially feasible way for PVC sterilization, while also providing a crucial reference for the further promotion and application of radiation sterilization technology.

## Materials and methods

### Model materials

The irradiated sample material is PVC. Common gamma radiation sources are Cr-51 source, Cs-137 source, Co-60 source, and K-40 source. The common reflective layer materials are aluminum, iron, lead, tungsten-nickel alloy, concrete, and water. The PVC sample holding platform is constructed using stainless steel material, while the walls of the irradiation workshop are built with concrete material. The material of the collimator is lead.

### Simulation parameters setup

The concrete factory building wall is set as cubes with a thickness of 12 cm to effectively prevent penetration of gamma rays^23^. The outer side length is 362 cm. Photon energies are set at 0.32 MeV (emission from Cr-51 decay), 0.662 MeV (emission from Cs-137 decay), 1.25 MeV (average energy from Co-60 decay^24^), and 1.461 MeV (emission from K-40 decay). The thickness of the reflective layer is set at 0, 1, 5, 8, 9, 10, 11, 12, 13, 14, 15, 16, 17, 18, 19, 20, 21, 22, 23, 24, and 25 cm. The model space is a cube with side lengths of 280, 285, 290, 295, 300, 305, 310, 315, 320, 325, and 330 cm.

The PVC material sample is set as a rectangular prism with a height of 15 cm, divided into three layers of 5 cm each: the top layer, middle layer, and bottom layer. To investigate the influence of the PVC sample area on energy deposition, we set the base area of the rectangular prism as 100 × 100 cm^2^ and 120 × 120 cm^2^. To study the effect of the distance between the radiation source and the PVC sample layer on the energy deposition of individual photons, the distance between the radiation source and the PVC sample layer is set at 150 cm and 159 cm. The densities of the model materials are shown in Table 1.

**Table 1:** Density of model materials.

Model material	Density (g/cm^3^)
Concrete	2.4
Tungsten-nickel alloy	18.4
Lead	11.34
Aluminum	2.7
Iron	7.8
Water	1
PVC	1.2

### Principle of radiation sterilization

The source photons emitted from the decay of the radiation source interact with the sample, leading to photons' energy transfer to electrons and generation scattered photons. Electrons further interact with the sample, depositing all their energy within the sample layer. Meanwhile, scattered photons undergo diversion during their transport through the model. Some scattered photons penetrate the model, while others deposit in the reflective layer. The remaining scattered photons reenter the sample layer, undergo collisions and ultimately deposit their energy within the irradiated sample through electron interactions.

In summary, during the process of irradiation sterilization, photons interact with the PVC sample layer, transferring energy to electrons. These electrons deposit energy within the PVC sample layer, leading to the bacterial cells, nucleic acids, proteins, and enzymes undergo excitation or ionization upon absorbing energy deposited by photons. Molecules in an excited state may experience bond cleavage, or react with other molecules and lead to the generation of free radicals. Or undergo ionization decomposition, and other molecular reactions, thereby resulting in the disruption of bacterial molecular structures. Furthermore, other essential molecules within bacterial cells may also absorb the deposited energy to undergo excitation or ionization. For instance, water may produce excited water molecules, electrons, and water ions, or undergo cleavage into hydrogen radicals and hydroxyl radicals, which initiate a series of oxidation–reduction reactions involving nucleic acids, proteins, and enzymes, ultimately leading to bacterial death^25,26^.

### MCNP simulation computational principles

The MCNP program is a Monte Carlo simulation calculation program for solving particle transport problems in complex geometric structures. Its simulation accuracy has been validated through numerous experiments. The program simulates the trajectories of emitted particles, records the partial energy depletion and generation of new particles upon their interaction within the sample, and continues tracking the new particles within the model until their complete disappearance.

**Figure 1:**
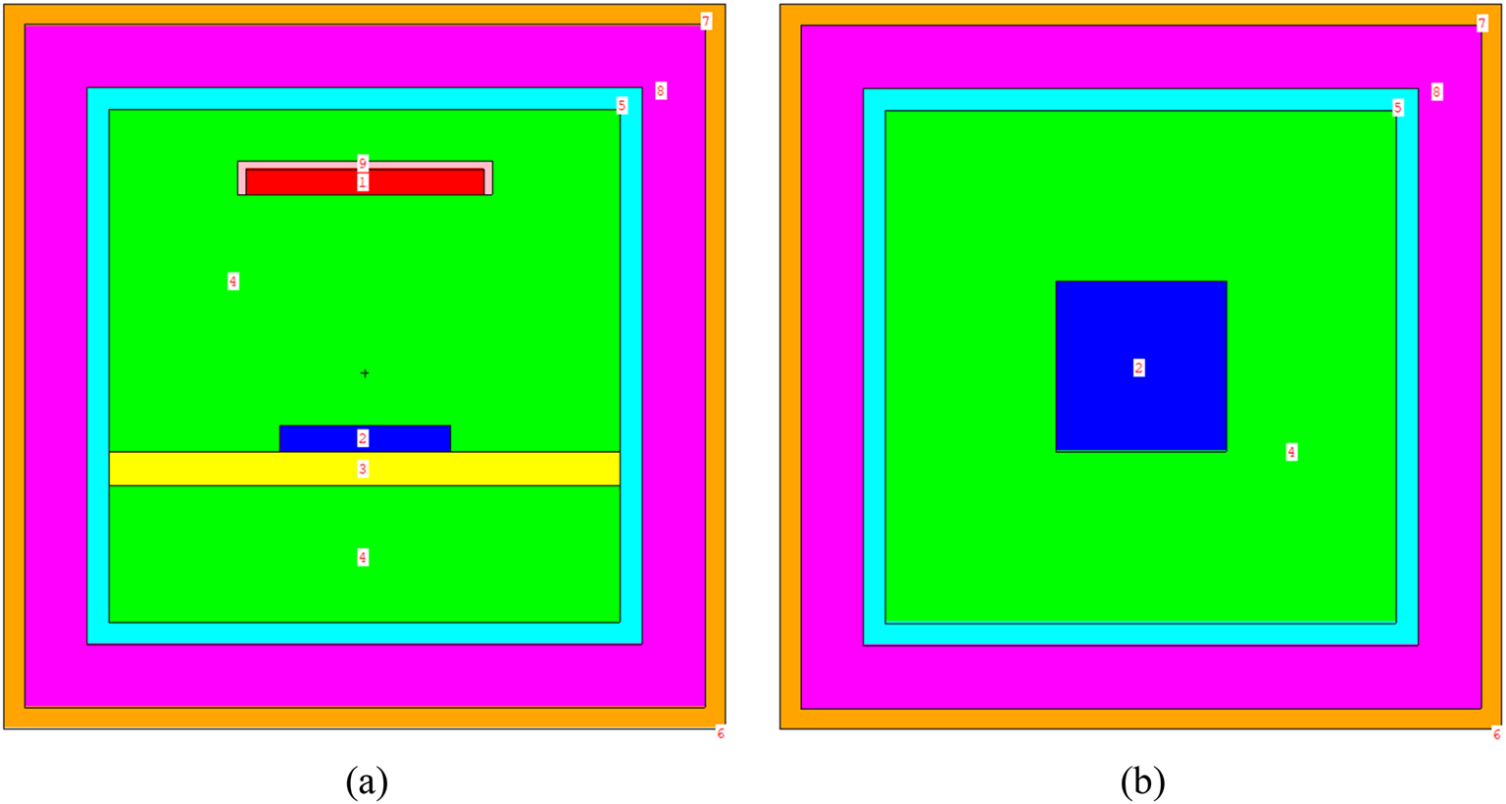
Radiation sterilization model. (**a**) Front view; (**b**) Plan view.

Based on the irradiation facility parameters, we utilize MCNP Visual Editor to obtain a schematic diagram of the model. The main view and top view of the radiation sterilization model are shown in Fig. 1a,b respectively.

In Fig. 1, Area 1 represents the radiation source, Area 2 represents the PVC sample layer, Area 3 represents the stainless steel containment platform, Areas 4 and 8 represent the air layers, Area 5 represents the reflective layer, Area 6 represents the outside of the facility (designated as a vacuum layer), and Area 7 represents the concrete walls. Area 9 represents the collimator.

**Figure 2:**
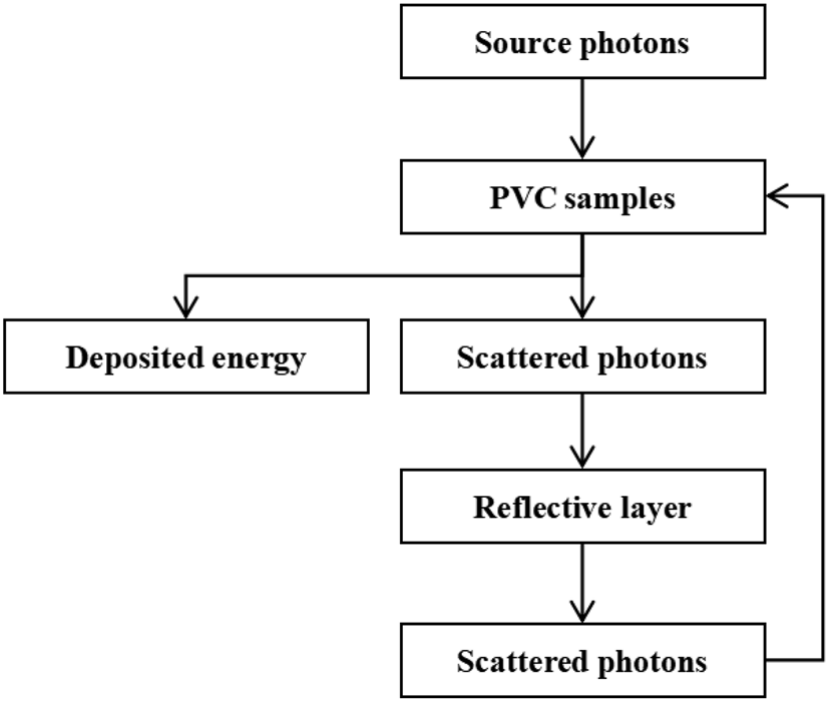
The movement trajectory of the source photon.

As source photons move within the irradiation sterilization model, they experience energy loss while traversing through the air. A portion of these photons enters the PVC sample and interact with it, depositing part of their energy while simultaneously generating scattered photons. Simultaneously, another fraction interacts with the stainless steel holding platform, depositing part of their energy while simultaneously generating scattered photons. During the movement of scattered photons within the model, branching occurs as well. Some of the scattered photons penetrate through the model after depositing a portion of their energy, while another portion continues transport, depositing energy and generating scattered photons until no new scattered photons are emitted. This entire process constitutes a complete trajectory of the source photon's movement. The simplified flowchart of the source photon's movement trajectory is illustrated in Fig. 2.

The MCNP program calculates the energy deposition using the F6 output card, where the energy deposition is calculated as follows^27–29^:

1 $${H}_{t}=\frac{{\rho }_{\alpha }}{m}\int dE\int dt\int d\Omega \times {\sigma }_{t}\left(E\right){\rm H}\left(E\right)\Psi \left(\overrightarrow{r}\right.,\Omega ,{\rm E},\left.t\right)$$

where ${\rho }_{\alpha }$ is atom density (atoms/barn-cm); $m$ is cell mass (g); ${\rm H}(E)$ is heating number (MeV/collision), ${\sigma }_{t}$ is microscopic total cross section (barns); $\Psi \left(\overrightarrow{r}\right.,\Omega ,{\rm E},\left.t\right)$ is angular flux familiar from nuclear reactor theory. $\Psi \left(\overrightarrow{r}\right.,\Omega ,{\rm E},\left.t\right)=\nu n\left(\overrightarrow{r}\right.,\Omega ,E,\left.t\right)$, where n is the particle density (particles/cm^3^/MeV/steradian) and $\upnu$ is velocity in cm/sh. Thus, the units of $\Psi$ are particles/cm^2^/sh/MeV/steradian (sh; 1 shake = 10^–8^ s).

In this process, the F6 tally card is used to track emitted photons. It records the energy of photons upon entering the sample, as well as the energy of scattered photons resulting from interactions within the sample. The energy difference between these two types of photons represents the initial energy deposition due to particle interactions (Formula 1).

The core data for MCNP in the article is derived from the ENDF/B-VI.8 evaluated data set. Specifically, the cross-section data for interactions between photons and materials are sourced from MCPLIB04 (id plib = ’04p’)^30^, while the cross-section data for interactions between electrons and materials are sourced from el03 (id elib = ’03e’)^31^.

## Results and discussion

Gamma rays entering the PVC sample layer undergo a series of interactions, primarily including the photoelectric effect, Compton scattering, and electron pair production. The predominant mode of interaction is influenced by factors such as the energy of the gamma photons, the material and thickness of the reflective layer, and the volume of model space. As the energy of the incident gamma photons increases, the energy deposited during interactions within the PVC sample layer also increases, causing the gamma rays to penetrate deeper into the sample layer and generating higher-energy scattered photons upon interaction. Scattered photons with greater energy, while continuing their transport within the model, contribute to larger energy depositions within the PVC sample layer and are positioned at greater depths. Furthermore, the distance between the radiation source and the PVC sample layer, along with the area of the irradiated PVC sample, also play a role in influencing the energy deposition of individual photons within the PVC sample layer. To investigate the impact of these factors on the single-photon energy deposition within the PVC sample layer, we conducted the following simulation^32^. We utilized statistical methods and employed Monte Carlo simulations to investigate the influence of the aforementioned irradiation facility parameters on the energy deposition in the PVC sample layer. We simulated a substantial amount of data and conducted a comprehensive analysis. The errors associated with each set of simulation results remained below 0.0005.

### The influence of gamma photon energy on individual photon energy deposition in PVC sample layer

**Figure 3:**
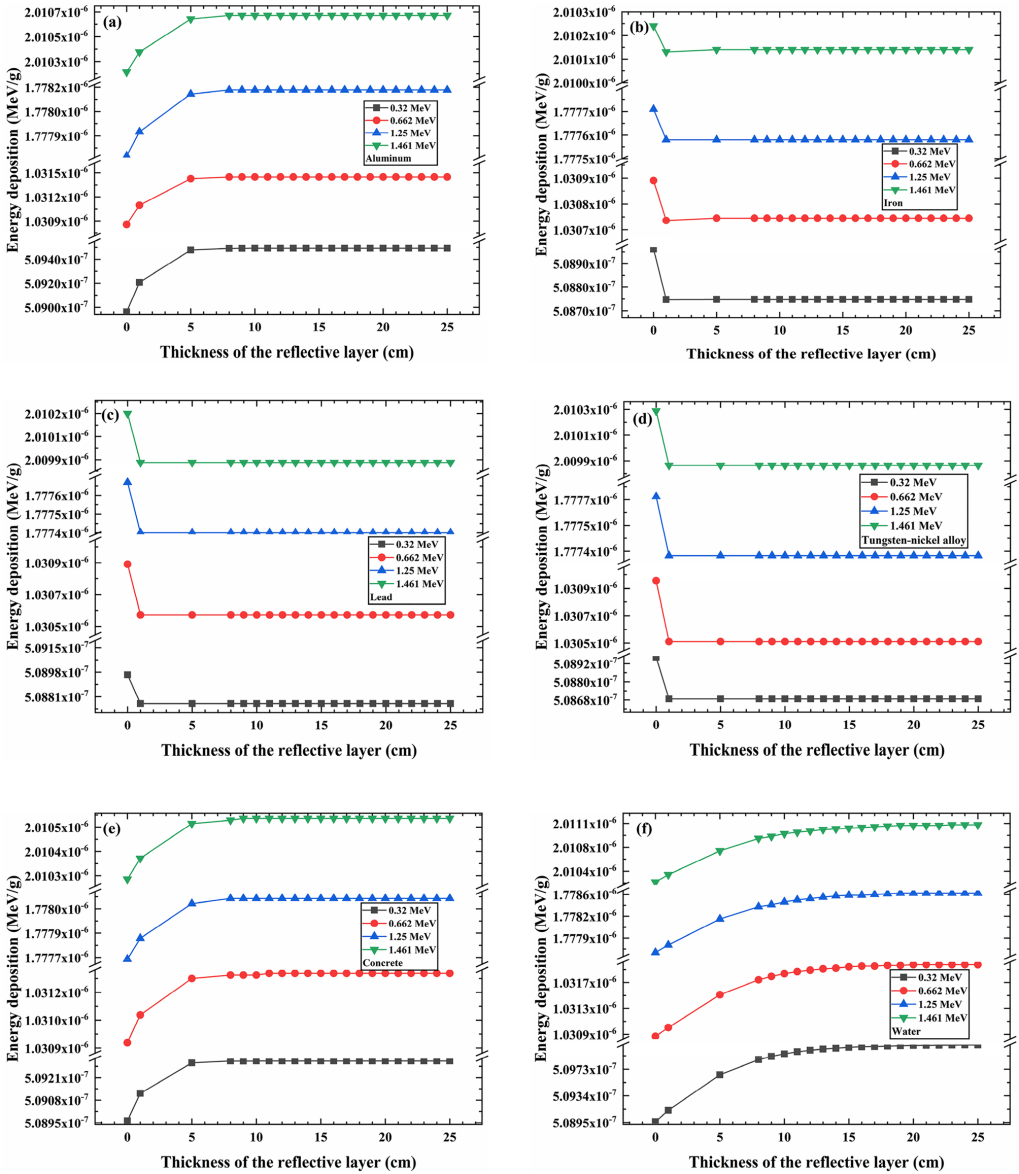
The individual photons energy deposition of different photon energies. Reflective layer materials are (**a**) aluminum, (**b**) iron, (**c**) lead, (**d**) tungsten-nickel alloy, (**e**) concrete, and (**f**) water.

Figure 3 illustrates the impact of reflective layer thickness on the individual photon energy deposition in the PVC sample layer for various photon energies.

The above results indicate that for the six reflective layer materials, the photon energy of 1.461 MeV (emitted from K-40 decay) demonstrates higher energy deposition in the sample layer. This confirms that higher photon energies lead to greater individual photon energy deposition in the sample layer. This is consistent with the results obtained by Arvind D and Hiroyuki Kadotani^33,34^.

It is crucial to highlight that K-40 decay has an extremely long half-life, low activity, very low natural abundance, and mainly releases β particles, making it unsuitable as a radiation source for our purposes^35^. Co-60 decay primarily emits gamma rays with energies of 1.17 MeV and 1.33 MeV. The average energy of these gamma rays is 1.25 MeV, and considering Co-60 has a longer half-life and better safety control, Co-60 is a suitable choice as the radiation source for the radiation device. Therefore, the Co-60 source is the best choice among the four mentioned radiation sources. The energy of the source photons is 1.25 MeV.

### The influence of model space volume on individual photon energy deposition in PVC sample layer

**Figure 4:**
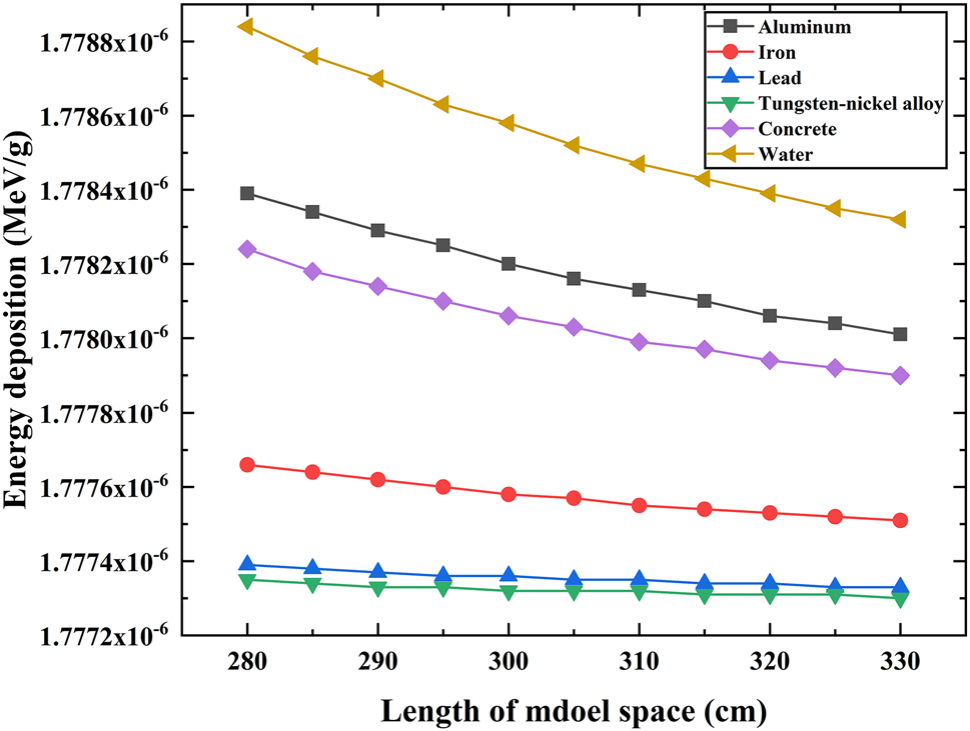
The individual photon energy deposition in different model space.

Figure 4 depicts the impact of model space volume on the individual photon energy deposition in the PVC sample layer. The results indicate that, across scenarios with six different reflective layer materials, with the model length increases, the individual photon energy deposition in the PVC sample layer gradually decreases. When the model side length approaches 330 cm, the variation in energy deposition becomes more gradual.

The reason for the above phenomenon is that as the model space increases, the energy loss of gamma photons in the air and the energy deposition in the reflective layer will increase during their transport within the model. Correspondingly, the energy deposition in the PVC sample layer will decrease. However, the proportion of energy deposition of scattered photons in the reflective layer is relatively small. With the increase of model space, the impact on energy deposition in the PVC sample layer is further reduced, leading to a leveling off of the variation in energy deposition in the PVC sample layer.

### The influence of reflective layer materials and thickness on the energy deposition of individual photons in the PVC sample layer

**Figure 5:**
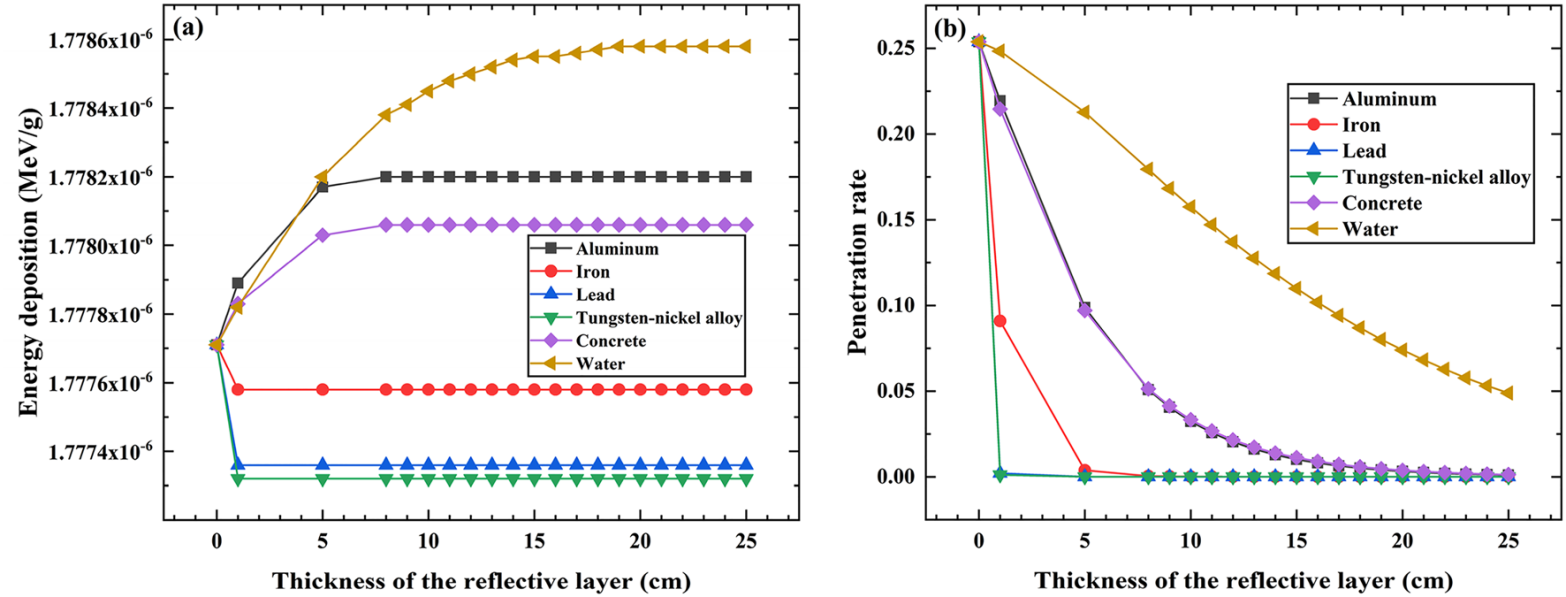
(**a**) The individual photon energy deposition of different reflective layer thickness. (**b**) The penetration rates of various reflective layer materials.

Figure 5a simulates the impact of reflective layer materials and thickness on the energy deposition of individual photons in the PVC sample layer. Figure 5a shows that when the reflective layer thickness is 0 cm (absence of reflective layer material), the energy deposition of individual photons in the PVC sample layer remains the same for all six different reflective layer materials. When iron, lead, and tungsten-nickel alloy are used as reflective layer materials, an increase in the reflective layer thickness leads to a decrease in the average energy deposition of individual photons in the PVC sample layer. This decrease becomes stable after the reflective layer thickness reaches 1 cm. However, when the reflective layer materials are changed to aluminum, concrete, or water, the average energy deposition of photons in the PVC sample layer exhibits a logarithmic increase with the increasing thickness of the reflective layer. This increase stabilizes once the reflective layer thickness reaches a certain point.

The primary reasons for the occurrence of the aforementioned phenomena are: the primary mode of interaction between radiation and matter is influenced by the energy of the radiation, the density of the material, and the atomic number^36^. With source photons having an average energy of 1.25 MeV and PVC density at 1.2 g/cm^3^, the primary interaction mode of interaction between the source photons and PVC sample is Compton scattering, resulting in the generation of numerous scattered photons. When the reflective layer material is absent (0 cm thickness), the 12 cm thick concrete wall will serve as the reflective layer. In this scenario, the primary mode of interaction between the scattered photons and the reflective layer material is Compton scattering, giving rise to new scattered photons that continue to propagate within the model until energy is deposited in the PVC sample layer. As the thickness of the reflective layer increases, different materials such as aluminum, iron, lead, tungsten-nickel alloy, concrete, and water are employed as reflective layer materials.

When the reflective layer materials are iron, lead, or tungsten-nickel alloy, because of the greater densities and atomic numbers of constituent elements compared to the concrete wall, the probability of the photoelectric effect is higher, followed by Compton scattering, and the electron pair production has the lowest probability. In the photoelectric effect process, scattered photons transfer all their energy to electrons, and no new scattered photons are generated. Compton scattering produces a smaller number of scattered photons, and electron pair production does not lead to scattered photons. Hence, the quantity of scattered photons is reduced, leading to a decrease in the deposited energy within the PVC sample layer. When the reflective layer reaches its saturation thickness, the energy deposition in the PVC sample layer will not change.

In contrast, when aluminum, or water are used as reflective layer materials, because of the smaller densities and atomic numbers of constituent elements compared to the concrete wall, the probability of the Compton scattering is higher. When scattered photons interact with the reflective layer, they undergo splitting, where some photons penetrate the reflective layer and exit the model, while others generate new scattered photons that continue to propagate within the model. Upon re-entering the sample layer, those new scattered photons interact again, resulting in energy deposition in the PVC sample layer. With an increase in reflective layer thickness, the number of photons penetrating the model gradually decreases, while the number of photons propagating within the model increases. Consequently, the energy deposition in the PVC sample layer increases. However, once the reflective layer reaches a saturation thickness, the number of split photons ceases to change, leading to a stabilization of energy deposition.

We employ the term "penetration rate" to represent the probability of photons escaping the model by passing through the reflective layer, expressed as the ratio of the number of photons escaping through the reflective layer to the total number of emitted photons. Figure 5b indicates that when the thickness of the reflective layer is 0 cm, the penetration rate is 0.25. The lower penetration rate is attributed to the fact that the majority of emitted source photons deposit their energy in the PVC sample and the stainless steel holding platform. Consequently, the resulting scattered photons pass through the model and exit. As the thickness of the reflective layer increases, the shielding effect of the reflective layer leads to a gradual reduction in the penetration rate until it reaches 0. During this process, the number of scattered photons moving within the model increases, resulting in a logarithmic increase in the energy deposition of photons in the sample layer. This trend stabilizes after the reflective layer reaches a certain thickness (saturation thickness). Subsequently, the energy deposition of individual photons in the sample layer no longer changes significantly with further increases in the reflective layer thickness.

The above results demonstrate that the choice of reflective layer material significantly influences the energy deposition efficiency of individual photons in the PVC sample layer^32,33,37^.Consequently, when water is used as the reflective layer material, it exhibits higher photon utilization efficiency and energy deposition. Aluminum and concrete follow closely in terms of energy deposition efficiency. However, water cannot encapsulate into a reflective layer with a thickness of 25 cm. In practical engineering applications, considering the strong penetrability of photons, a balance between safety and photon energy deposition efficiency, concrete, and aluminum are more suitable choices as reflective layer materials. Therefore, in the subsequent analysis, we will focus on simulations using aluminum and concrete materials.

### The influence of PVC sample area on the energy deposition of individual photons

**Figure 6:**
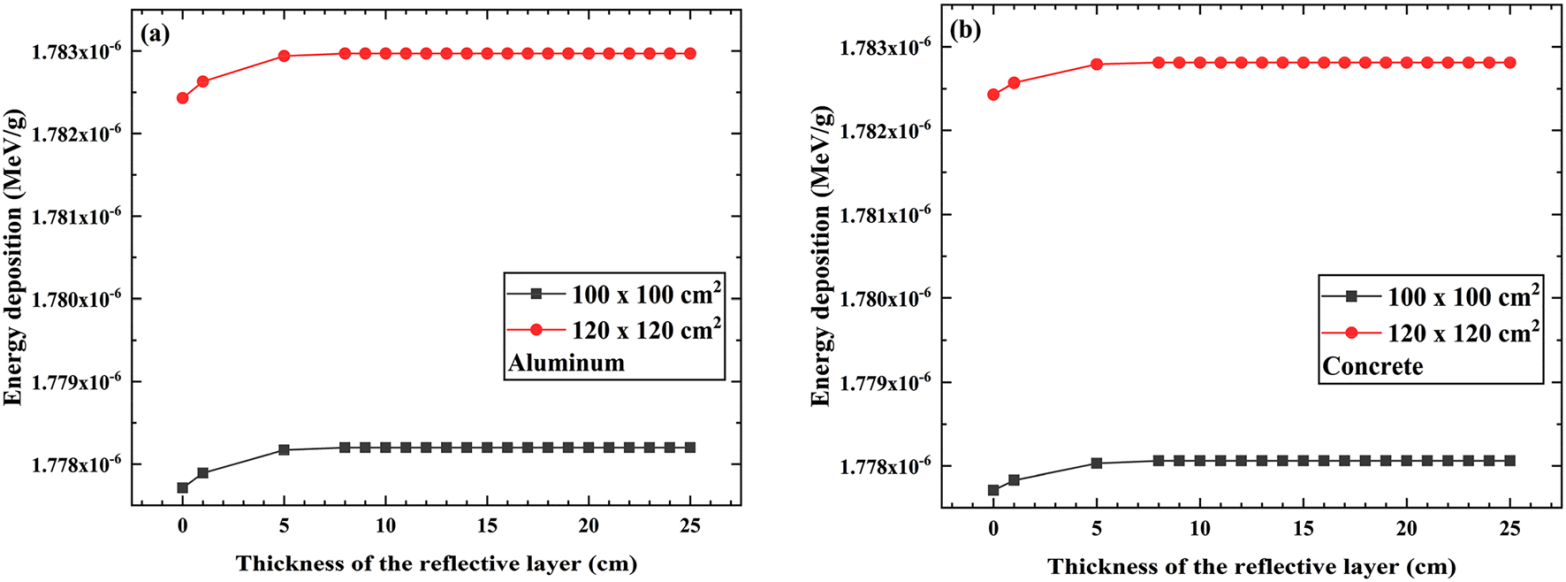
The energy deposition for different PVC sample areas. (**a**) Reflective layer material is aluminum, (**b**) Reflective layer material is concrete.

Figure 6 simulates the effect of the PVC sample layer area on the energy deposition of individual photons in the PVC sample layer when the reflective layer materials are aluminum and concrete.

The results indicate that the energy deposition of individual photons in the PVC sample layer is greater for an area of 100 × 100 cm^2^ compared to 120 × 120 cm^2^. This is because a larger PVC sample layer area increases the probability of photon collisions upon entering the PVC sample layer, leading to a higher likelihood of energy deposition within the sample layer. As a result, when the PVC sample layer area is increased, the energy deposition in the PVC sample layer also increases.

However, in the simulation calculations using the MCNP program for the energy deposition of individual photons in the PVC sample layer, the process involves first calculating the energy deposited within the PVC sample layer, followed by calculating the mass of the PVC sample layer. The ratio of energy deposition to mass in the PVC sample layer represents the average energy deposition of a single photon within the PVC sample layer. When the PVC sample layer thickness remains constant, a larger area implies a larger mass, which leads to a smaller energy deposition per unit mass. This means that the energy deposition of individual photons in the PVC sample layer is inversely correlated with the sample layer area^19^.

Hence, it can be concluded that increasing the PVC sample layer area does not necessarily increase the energy deposition of individual photons within the PVC sample layer.

### The influence of the distance between the radiation source and the PVC sample layer on the energy deposition of individual photons

**Figure 7:**
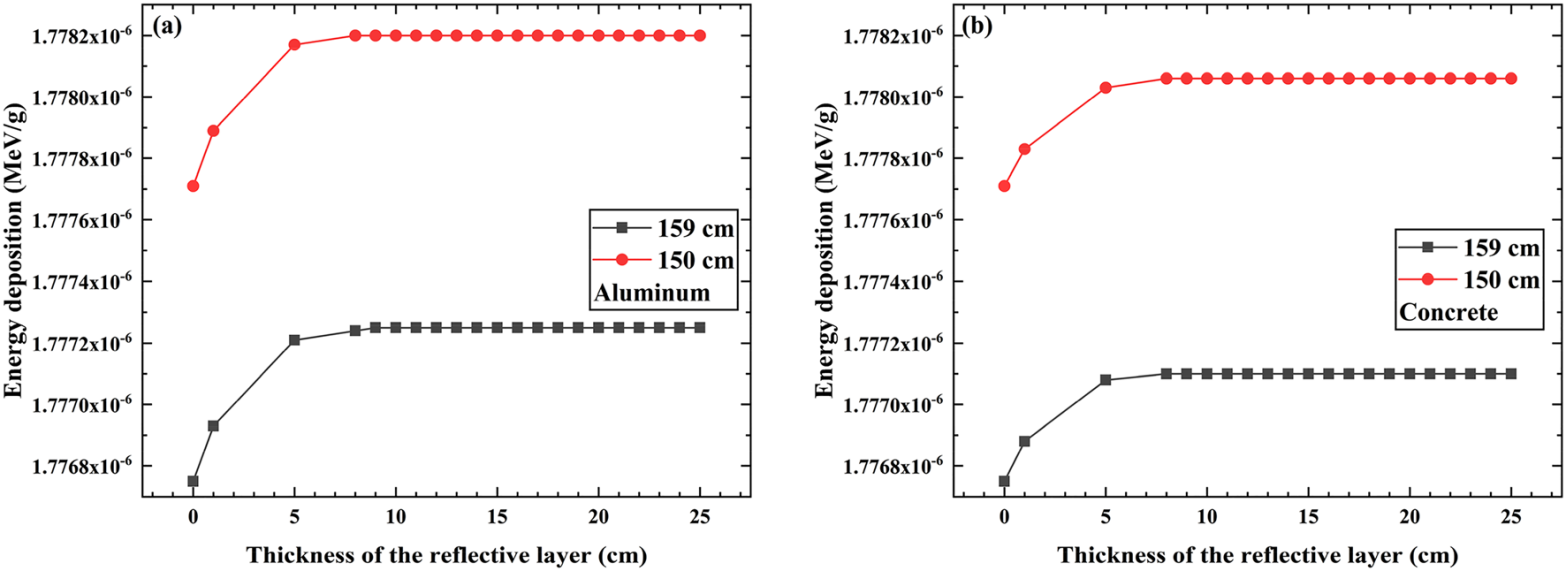
The energy deposition in the PVC sample layer with the different distance between the radiation source and the PVC sample layer, respectively. (**a**) The reflective layer material is aluminum, (**b**) The reflective layer material is concrete.

When the radioactive source decays and emits gamma rays, these gamma rays also undergo interactions with the air during their transport within the model, leading to energy loss. As the distance between the radiation source and the PVC sample layer increases, the probability of collisions of gamma rays with air during their movement in the air also increases, resulting in greater energy loss. Therefore, the distance between the radiation source and the PVC sample layer also affects the energy deposition of individual photons. Figure 7 simulates the effect of the distance between the radiation source and the PVC sample layer on the energy deposition of individual photons.

The simulation results indicate that when the distance between the radiation source and the PVC sample layer is 159 cm, the energy deposition of individual photons is smaller compared to the distance of 150 cm. From this, it can be inferred that the energy deposition of individual photons in the PVC sample layer is negatively correlated with the distance from the radiation source to the PVC sample layer. As the distance increases, the energy deposition in the PVC sample layer will decrease. This is consistent with the findings by Fang Liu^19^.

### The influence of PVC sample thickness on the energy deposition of individual photons

**Figure 8:**
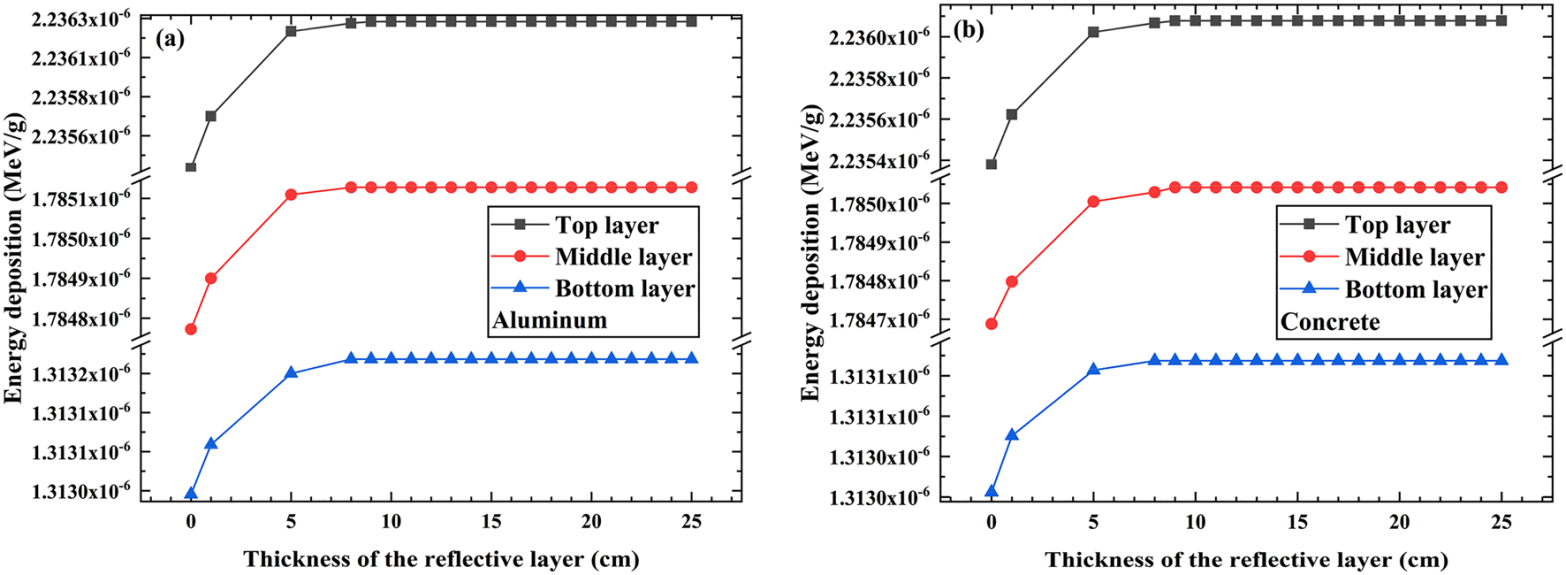
The energy deposition in the top, middle, and bottom layer of the PVC sample. (**a**) Reflective layer material is aluminum, (**b**) Reflective layer material is concrete.

The energy deposition of individual photons in different layers of the PVC sample can also vary. To investigate the influence of PVC sample thickness on the energy deposition of individual photons, we divided a 15 cm thick PVC sample layer into three equal layers: the top layer with a thickness of 5 cm, the middle layer with a thickness of 5 cm, and the bottom layer with a thickness of 5 cm. The simulation results are shown in Fig. 8.

Figure 8 shows that among the three aforementioned regions, the top layer has the highest photon energy deposition, followed by the middle layer, and the bottom layer exhibits the lowest energy deposition. This is attributed to the higher energy of gamma photons emitted from the Co-60 source decay. When these photons collide with the PVC sample, they transfer energy to electrons. Electrons have weaker penetration, leading to greater energy deposition in the top layer. Scattered photons, which lose some energy, continue to penetrate deeper into the sample and deposit energy in the middle and bottom layers.

As the interaction progresses, the energy of scattered photons diminishes, leading to fewer scattered photons entering the middle and bottom layers, resulting in less energy deposition in the bottom layer. To ensure effective inactivation of bacteria and viruses in the 15 cm-thick PVC sample layer, it is crucial to ensure sufficient inactivation of microorganisms in the bottom layer. Therefore, the optimal measurement for gamma photon energy deposition in the PVC sample layer is based on the energy deposition in the bottom layer.

### Estimating radiation sterilization time.

The survival rate of bacteria is influenced by the sterilization dose. The bacterial survival curve indicates that as the radiation dose increases, the survival rate of bacteria gradually decreases^38^. The International Atomic Energy Agency (IAEA) has recommended a standard sterilization dose of 25 kGy when the contamination level and type of contaminating microorganisms cannot be confirmed^39,40^. IAEA also recommends an irradiation sterilization dosage of 25 kGy for medical devices^41^. Therefore, referencing a dose of 25 kGy as the lethal dose for bacteria and viruses in PVC samples is reasonable.

Due to the weight factor of 1 for gamma rays, the radiation sterilization dose for PVC samples is:

2 $$ {\text{D}} = {25}\;{\text{kGy}} $$

The intensity of gamma photons depends on the activity of the radiation source. According to the classification of radiation sources in the radiation device, the activity of the Co-60 source in the radiation device is 1.5 × 10^5^ TBq^42^. Co-60 source releases 2 gamma photons per decay, which allows us to calculate the intensity of photons as follows:

3 $$\mathrm{A}=1.5\times {10}^{5}\times 2\times {10}^{12}=3\times {10}^{17 }(\mathrm{n}/\mathrm{s})$$

By optimizing the parameters of the model radiation facility, the higher energy deposition in the bottom layer of the PVC sample is observed, measuring 1.31320 × 10^–6^ MeV/g (reflective layer material is aluminum) and 1.31315 × 10^–6^ MeV/g (reflective layer material is concrete).

The simulated radiation sterilization time for PVC samples is^43^:

4 $$t=\frac{D(Gy)}{d(Mev/g)\times {10}^{6}\times 1.6\times {10}^{-19}\times {10}^{3}\times Q\times A(n/s)\times 60} (\mathrm{Minutes})$$

where, d represents the individual photon energy deposition in the PVC sample layer, D is the sterilization dose, Q is the weight factor, and A is the photon intensity.

By substituting the data, the calculated radiation time for PVC samples is approximately 6.61 min when the reflective layer materials are aluminum and concrete. Considering the cost factor, concrete is much more cost-effective than aluminum. Hence, from a practical engineering perspective, choosing concrete as the reflective layer material is more preferable.

The use of radiation sterilization techniques significantly enhances the sterilization efficiency of PVC materials and concurrently explores a viable avenue for recycling irradiated PVC materials. Nevertheless, elevated radiation doses may exert adverse effects on the stability of PVC materials, necessitating stringent control over the radiation dosage. The references 42 and 43 findings indicate that adding the vegetable oil extracted from coffee grounds (OGC) to PVC materials significantly enhances their stability, preventing degradation in low-dose irradiation scenarios. This has the potential to increase the recyclability of PVC materials; however, extensive experimental validation is still required to confirm these outcomes^44,45^.

## Conclusion

The research findings indicate that the energy deposition of individual photons in the PVC sample layer is influenced by multiple factors including photon energy, spatial dimensions of the model, reflective layer material, reflective layer thickness, irradiated PVC sample layer area, distance between the radiation source and the PVC sample layer, and sample layer thickness.

In summary, compared with the sterilization time of 20–90 min from medical waste high-temperature steam sterilization^46^, by optimizing the parameters of the model radiation facility, the irradiation sterilization time can be reduced to 6.61 min according to the radiation sterilization dosage standards. At this time, the photon energy is 1.25 MeV, the model space is 300 × 300 × 300 cm^3^, the reflective layer material is concrete and the thickness is 8 cm, the PVC sample layer area is 100 × 100 cm^2^, the distance between the radiation source and the PVC sample layer is 150 cm, the energy deposition in the bottom layer of the PVC sample layer is 1.31315 × 10^–6^ MeV/g. This study offers a potentially feasible way for PVC sterilization, while also providing a crucial reference for the further promotion and application of radiation sterilization technology.

## Data Availability

Original data are available from the corresponding author upon reasonable request.
